# Functional Foods Based on Postbiotics as a Food Allergy Treatment

**DOI:** 10.3390/foods14203584

**Published:** 2025-10-21

**Authors:** Nader Khani, Faezeh Shirkhan, Mansour Rabie Ashkezary, Vahideh Sarabi Aghdam, Roya Abedi Soleimani, Seyed Mohamad Javad Shokouhian, Negin Hosseinzadeh, Aziz Homayouni-Rad

**Affiliations:** 1 Student Research Committee, Tabriz University of Medical Sciences, Tabriz 5166614766, Iran; naderxani1996m@gmail.com; 2 Department of Food Science and Technology, Faculty of Pharmacy, Tehran Medical Sciences Islamic, Tehran 676533314, Iran; faezehaut@gmail.com; 3 Department of Agricultural, Food and Forest Sciences (SAAF), University of Palermo, Viale delle Scienze Bldg. 5, Ent. C, 90128 Palermo, Italy; mansourrabie@gmail.com; 4 Department of Food Science and Technology, Faculty of Nutrition & Food Sciences, Nutrition Research Center, Tabriz University of Medical Sciences, Tabriz 5166614766, Iran; vahidehsarabi@gmail.com (V.S.A.); roya_abedi@ymail.com (R.A.S.); hosseinzadeh@tbzmed.ac.ir (N.H.); 5 Faculty of Veterinary Medicine, Karaj Branch, Islamic Azad University, Karaj 3149968111, Iran; mohammadjavad@gmail.com

**Keywords:** food allergy, functional food, postbiotic, probiotic, immune system

## Abstract

Functional foods are defined as consumables that, in addition to providing essential nutrients, exhibit health-promoting properties when consumed regularly over a long period of time. Globally, there has been great emphasis on their consumption as a cost-effective and safer alternative for the prevention, treatment, and management of various health-related issues, including food allergies. Over the past two decades, the prevalence and severity of clinical manifestations of food allergy (FA) have increased significantly. FA results from the breakdown of immune tolerance. There are different types of functional foods that can be effective in the treatment of FA. Functional foods based on postbiotics that are produced through fermentation include kefir, yogurt, cheese, and fermented milk. This review highlights the potential role of functional foods based on postbiotics in the treatment of FA.

## 1. Introduction

FA is acknowledged as the most prevalent immune disorder, arising from a disruption of immune tolerance. FA is regarded as a significant global health concern that adversely affects the quality of life of individuals, particularly in industrialized nations. Over the past two decades, there has been a notable increase in both the prevalence and the severity of clinical manifestations associated with FA, which entails considerable health and economic ramifications, including an upsurge in medical consultations, treatments, and additional financial burdens for affected individuals, especially within the pediatric demographic. Research findings indicate that over 170 distinct food items have been implicated in the onset of FA, with the more severe allergic reactions commonly linked to the ingestion of fish, shellfish, peanuts, milk, tree nuts, soy, wheat, seeds, and eggs, with variations observed among individuals, nations, and geographical regions [1]. The principal risk factors contributing to the development of FA are associated with genetic predispositions, environmental influences, and their interactions, leading to a compromise in immune tolerance. Conversely, there is a growing body of evidence indicating that the population dynamics and composition of gut microbiota play a pivotal role in immunological maturation and are recognized as critical determinants in the modulation of food-allergic responses. Therefore, maintaining gut microbiota eubiosis, particularly during the early stages of life, may significantly impact the progression of FA [2].

The engagement of the pharmaceutical sector with natural products has experienced a notable augmentation, owing to their inherent chemical and biological heterogeneity, which facilitates their application in the management of various diseases. Consequently, they are regarded as a viable alternative to synthetic pharmaceuticals. Within this context of natural products, functional foods have been prominently acknowledged over numerous years as a potentially advantageous component in the therapeutic approach to diverse diseases. Functional foods are delineated as products that offer health advantages, as well as enhancements in physical and mental well-being, that transcend basic nutritional provision. Hence, the incorporation of functional foods is perceived as an efficacious and economically sustainable methodology for health maintenance and the reduction of pharmaceutical-related expenditures [3]. Postbiotics represent one of the most significant functional constituents investigated in the research pertaining to FA management. The designation postbiotic refers to the utilization of non-viable cellular entities or cellular fractions, which, when administered in sufficient quantities, provide a health benefit to the host organism (Table 1). This review emphasizes the definition of postbiotics and the prospective importance of postbiotic-enriched functional foods in the management of food allergies.

## 2. Definition of Postbiotics

The designation postbiotics has recently been confined to metabolites and cell-free supernatants (CFSs), along with soluble factors, which encompass both products and metabolic byproducts secreted by living bacteria [14,15,16,17]. The term “postbiotics” refers to the structural components of probiotic microorganisms, their metabolites, and/or signaling molecules that possess a specific chemical structure capable of enhancing host-specific physiological functions, as well as regulatory, metabolic, and behavioral responses associated with the actions of the host’s indigenous microbiota [18]. These compounds can be found in a variety of fermented foods, such as kefir, kimchi, sauerkraut, tempeh, yogurt, and certain pickles, in addition to their presence within the human body [19]. The primary categories of postbiotics include organic acids, short-chain fatty acids (SCFAs), Lipoteichoic Acid (LTA), exopolysaccharides (EPSs), and bacteriocins. The benefits associated with the use of postbiotics can be categorized into direct and indirect effects [20]. Direct benefits result from the interaction between postbiotics and host cells, while indirect benefits involve the promotion of the growth of health-promoting microbial strains and the inhibition of harmful strains. The effects of postbiotics can vary significantly based on the specific microorganism, strain, and the metabolic products produced, similar to the variability observed with prebiotics [21]. Importantly, the most pronounced beneficial effects of postbiotics are their antimicrobial, antioxidant and anticancer effects, anti-inflammatory, lowering blood cholesterol, anti-obesity, and modulating the immune system [22]. Postbiotics have been chosen as the main component of functional foods due to their health-promoting effects.

## 3. Functional Foods Based on Postbiotics

Functional foods are defined as consumables that confer health advantages extending beyond mere nutritional value due to their incorporation of bioactive constituents, which may enhance overall health or mitigate the incidence of specific diseases [23]. In contrast to conventional foods that predominantly deliver macronutrients and micronutrients, functional foods are augmented with components such as probiotics, prebiotics, omega-3 fatty acids, antioxidants, vitamins, minerals, or phytochemicals derived from plants, all of which may positively affect physiological functions [24]. For instance, yogurt abundant in probiotics can foster gastrointestinal health by harmonizing intestinal microbiota, whereas foods enriched with omega-3 fatty acids, including certain margarine products or eggs, can promote cardiovascular and cognitive health. Likewise, whole grains, nuts, and berries are replete with antioxidants and dietary fiber that contribute to the reduction in inflammation and the enhancement of digestive health [25]. For instance, yogurt abundant in probiotics can foster gastrointestinal health by harmonizing intestinal microbiota, whereas foods enriched with omega-3 fatty acids, including certain margarine products or eggs, can promote cardiovascular and cognitive health. Likewise, whole grains, nuts, and berries are replete with antioxidants and dietary fiber that contribute to the reduction in inflammation and the enhancement of digestive health [26]. The concept of functional foods bridges the gap between nutrition and medicine, showing that diet can play an active role in preventing chronic illnesses such as cardiovascular disease, diabetes, and even some cancers. As consumer awareness about wellness grows, the demand for functional foods has expanded globally, driving innovation in the food industry to create products that not only taste good but also support long-term health. However, it is important to note that functional foods are not substitutes for a balanced diet or medical treatment; rather, they are complementary choices that, when included as part of a healthy lifestyle, can help individuals maintain better physical and mental well-being [27,28].

The augmented understanding of functional foods has precipitated the emergence of a novel cadre of health-related products, inclusive of formulations that incorporate probiotics. Nevertheless, a significant concern pertaining to the utilization of probiotics is the presence of antibiotic resistance genes within certain probiotic strains, which possess the capacity to transfer these antibiotic resistance genes to pathogenic bacteria via horizontal gene transfer [29]. An additional predominant issue linked to the formulations of probiotic products (i.e., pharmaceutical and commercial food-based offerings) is the preservation of bacterial viability throughout the processes of product manufacturing and storage, as the viability of probiotic organisms within a delivery system (i.e., pharmaceutical formulations and commercial food-based products) may be influenced by various factors, including interactions with concomitant microbial species, the final acidity of the product, water activity, temperature, nutrient availability, growth promoters and inhibitors, inoculation levels, fermentation duration, dissolved oxygen levels, and formulation techniques such as freeze drying, spray drying, or freeze concentration [30]. Furthermore, inconsistencies between the declared and actual probiotic concentrations in commercial products intended for both human and veterinary applications have been documented in prior studies [31]. Consequently, this instability in probiotic efficacy may undermine the anticipated health advantages associated with probiotic products [32]. Conversely, postbiotics exhibit greater stability compared to the live bacteria from which they are derived, demonstrate water solubility, and maintain activity across a broad pH spectrum, thereby facilitating their incorporation into a diverse array of food products [33]. Postbiotic-based functional foods are an emerging category designed to provide health benefits through bioactive compounds produced by probiotic microorganisms, without the need for live bacteria. These foods contain metabolites that have been shown to support gut health, modulate immunity, and provide antioxidant and antimicrobial effects. Unlike probiotics, postbiotic-based functional foods are more stable and safer for people with weakened immune systems, and can be incorporated into a wide range of products such as dairy products, beverages, and supplements, and used as a tool in the treatment of food allergies.

## 4. Postbiotics in FA Treatment

Their therapeutic efficacy resides in their capacity to modulate the immune response, augment intestinal barrier integrity, and mitigate inflammatory processes. Empirical research has demonstrated that specific postbiotic entities can facilitate the maturation of regulatory T cells and inhibit Th2-mediated responses, which are pivotal in the etiology of allergic manifestations [34,35]. Through the modulation of gut microbiota and immune signaling cascades, postbiotics present a novel and potentially efficacious approach for the management of food allergies. The biological functions of distinct postbiotics in the context of FA treatment encompass the following [36,37,38].

### 4.1. Bacteriocins

Bacteriocins can indirectly regulate immune responses in food allergy by modulating the gut microbiota composition. By selectively inhibiting pathogenic or opportunistic microbes, bacteriocins help maintain a balanced microbial ecosystem that favors the growth of beneficial bacteria capable of producing anti-inflammatory metabolites such as short-chain fatty acids. This healthier microbial environment strengthens the intestinal barrier, reduces allergen translocation, and promotes the expansion of regulatory T cells, which are crucial for maintaining oral tolerance. Consequently, bacteriocins do not act directly on immune cells but create favorable conditions within the gut that shape immune signaling, dampen Th2-driven allergic responses, and contribute to reduced hypersensitivity to food allergens.

Bacteriocins, which are hydrophobic antimicrobial peptides synthesized by ribosomes, play an essential role in suppressing the growth of intestinal infections and preserving the homeostasis of the human bacterial community. Bacteriocins synthesized by Gram-negative bacteria typically comprise proteins of higher molecular weight (for instance, bacteriocins derived from *Escherichia coli* range from 449 to 629 amino acids), whereas those originating from Gram-positive bacteria are generally peptides consisting of fewer than 70 amino acids. The generation of bacteriocins is frequently regarded as a characteristic of probiotic organisms [39]. Bacteriocins provide a competitive advantage to probiotics, facilitating their colonization within the human ecosystem and thwarting the encroachment of rival bacterial species into the host microbiota. Typically, bacteriocins demonstrate a specific narrow-spectrum activity against pathogenic microorganisms, exerting minimal influence on the composition of the commensal microbiota. Bacteriocins synthesized by microorganisms present in consumed foods (notably those that are fermented) contribute to the survival and establishment of probiotics within gut microbial communities [40]. Based on the findings of various studies, allergic conditions such as FA are closely linked to the gut microbiota of the host, as inflammatory responses manifest at the epidermal interface. The gut microbiota plays a significant role in the digestive process and the defense against the incursion of pathogenic microorganisms. Moreover, it constitutes the primary assembly of microbial-derived biomolecules that possess substantial potential to enhance epithelial barrier integrity and to elicit an appropriate immunological response [41]. A multitude of factors including genetics, environmental influences, dietary habits, psychosocial stressors, medication usage, infections, general health, and lifestyle choices can influence the composition of the gut microbiota [42]. Epidemiological data, alongside the outcomes of in vivo studies, have illustrated that alterations in the gut microbial composition and/or equilibrium can precipitate the emergence of dysbiosis, particularly during early developmental stages, which is subsequently correlated with immune dysfunction and the proliferation of allergic conditions [43]. In this context, research utilizing a murine model has demonstrated that disruption of microbial homeostasis directly influences the onset of food allergies [44]. The composition and/or balance of the host’s gut microbial communities exhibit considerable plasticity during the initial 1000 days of life and infancy, and continue to be rapidly modified in response to environmental perturbations, including the colonization by pathogenic microbes and their subsequent invasion into the bloodstream and tissues, antibiotic treatments, and dietary factors, which can consequently disrupt immune homeostasis [45]. In this regard, epidemiological evidence associates prenatal and early postnatal antibiotic treatments with a later onset of cow’s milk allergy. Conversely, the findings from an analysis of a prospective birth cohort corroborate that gut microbial imbalances (dysbiosis) during early life (the first 100 days) can significantly influence inflammatory responses and the development of allergic diseases [46]. Thus, the gut microbiota of the host serves multiple roles in the initiation, modulation, and amplification of allergic sensitization [47].

**Figure 1:**
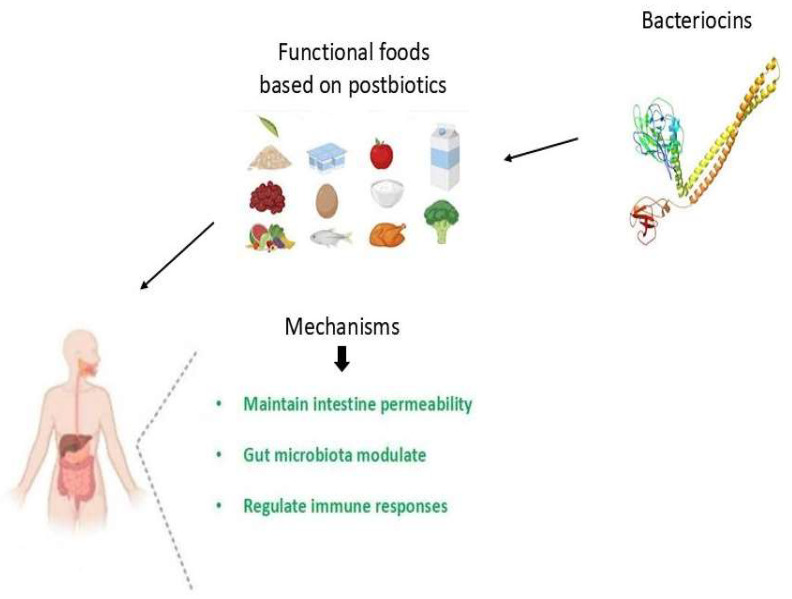
The mechanisms of bacteriocins in food allergies.

On the contrary, an individual exhibiting dysbiosis within the gastrointestinal tract experiences a modification of the predominant microbial community in the intestine, favoring pathogenic microorganisms. The proliferation of this microbial population, coupled with their colonization of epithelial cell surfaces, effectively diminishes the growth potential of beneficial microbiota, in addition to the production of secondary metabolites, including toxins, which exert deleterious effects on the host’s health status. The ingestion of pharmaceutical and/or functional food products containing postbiotic compounds is anticipated to primarily inhibit and ultimately cease the proliferation of pathogenic microorganisms, thereby leading to a reduction in the synthesis of associated toxins [44]. In this regard, the most significant antimicrobial mechanism attributed to postbiotics is due to the presence of compounds such as bacteriocins (Figure 1) [48]. Bacteriocins have the capacity to indirectly modulate immune responses associated with food allergies through the alteration of gut microbiota composition. By selectively targeting and inhibiting pathogenic or opportunistic microorganisms, bacteriocins contribute to the preservation of a balanced microbial ecosystem that is conducive to the proliferation of beneficial bacteria, which in turn are capable of synthesizing anti-inflammatory metabolites such as short-chain fatty acids. This enhanced microbial milieu fortifies the intestinal barrier, mitigates allergen translocation, and fosters the proliferation of regulatory T cells, which are essential for the maintenance of oral tolerance. Consequently, it is plausible that bacteriocins do not exert direct effects on immune cells; rather, they establish favorable conditions within the gut that influence immune signaling, modulate Th2-mediated allergic responses, and assist in diminishing sensitivity to food allergens [49].

### 4.2. SCFAs

SCFAs represent the most extensively investigated category of postbiotics. The gut microbiota synthesizes SCFAs through the fermentation of plant-derived polysaccharides, including oligofructose and inulin, resulting in the formation of respective fatty acid salts (i.e., acetate, propionate, and butyrate) [50]. SCFAs play a crucial role as metabolites essential for the preservation of intestinal homeostasis. Specifically, SCFAs contribute to the reduction in luminal pH, thereby inhibiting the proliferation of pathogenic bacteria. Each SCFA possesses a unique molecular structure, leading to distinct influences on human health. Butyrate, recognized as the most extensively studied SCFA, serves as the principal energy substrate for the proliferation of epithelial cells and plays a vital role in the regeneration of intestinal epithelial cells [51]. The production of butyrate is primarily facilitated by intestinal bacteria, predominantly *Enterococcus faecalis* and *Enterococcus rectus*, through pathways such as CoA transferase or butyrate kinase.

**Figure 2:**
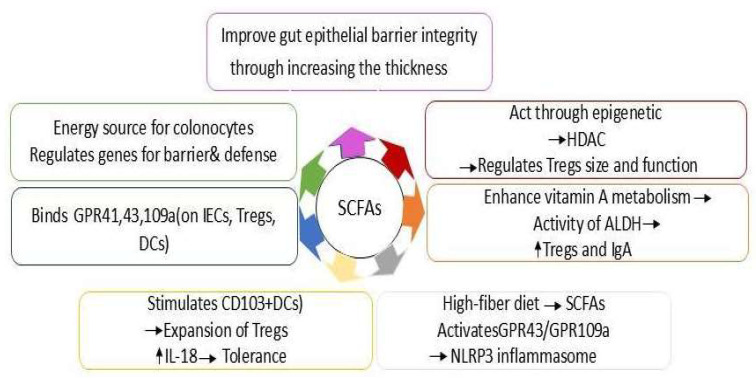
The main mechanisms of SCFAs against food allergies.

SCFAs ignite both immune and non-immune defenses against food allergies. They serve as the primary fuel for colonocytes, shaping the expression of genes crucial for the permeability and defense mechanisms of the gut epithelial barrier. When gut epithelial barrier permeability rises, it leads to an increased antigen absorption, fostering a Th2-type allergic reaction by activating ILC2s, mast cells, basophils, and dendritic cells. SCFAs enhance the integrity of the gut epithelial barrier by thickening the mucus layer (boosting the expression of mucin genes, especially MUC2) and augmenting the expression of tight junctions [52]. Within the realm of immune action, SCFAs operate through various pathways. One of the molecular pathways through which SCFAs influence immune system activities involves binding to specific G protein-coupled receptors (GPRs) such as GPR43, GPR41, and GPR109a. These receptors are not only found on intestinal epithelial cells (IECs) but also on gut immune cells like Tregs and dendritic cells. SCFAs influence gut CD103+ dendritic cells by activating the GPR109a receptor on their surface, enabling this tolerogenic subpopulation of dendritic cells to stimulate the proliferation and expansion of Tregs in the mesenteric lymph nodes [53]. Additionally, the GPR109a receptor facilitates the SCFAs-induced production of IL-18 in the colonic epithelium, which is crucial for bolstering tolerance to commensal bacteria and fostering gut homeostasis. SCFAs also enhance the metabolism of vitamin A, which in turn ramps up the activity of aldehyde dehydrogenases (ALDHs) in gut CD103+ dendritic cells, leading to an increase in Tregs and the production of IgA [54]. Furthermore, a high-fiber diet-induced activation of GPR43 and GPR109A sparks the NLRP3 inflammasome, which is vital for maintaining gut homeostasis. SCFAs are capable of influencing through epigenetic means as well [55]. They can pass through the cell membrane, thus inhibiting histone deacetylases (HDACs) in epithelial and gut immune cells. The acetylation/deacetylation of histones represents an epigenetic mechanism that alters cellular gene expression without changing the genomic DNA sequence [56]. The downstream epigenetic impact of SCFAs on enterocytes manifests through the regulation of genes tied to energy metabolism, cellular proliferation and differentiation, and bolstering gut barrier integrity by increasing the expression of tight junctions and thickening the mucus layer [57]. By inhibiting HDAC, SCFAs also influence the size and functionality of Tregs in the colon. In fact, HDAC inhibition resulted in an uptick in FOXP3 expression and Tregs numbers, enhancing the suppressive capabilities of FoxP3+ Tregs under homeostatic conditions and amplifying Tregs’ cell-mediated relief of colitis in mice. Through HDAC inhibition, SCFAs can also spur B cell differentiation and the synthesis of IgA and IgG (Figure 2) [58,59,60]. The research conducted by Tan et al. 2016 demonstrated that the oral administration of dietary fiber and SCFA postbiotics (notably acetate and butyrate) enhances oral tolerance and concurrently mitigates food (peanut) allergy in a murine model by augmenting retinal dehydrogenase activity in CD103þ dendritic cells, which is significantly reliant on vitamin A metabolism and enhances IgA responses [61]. Furthermore, a study executed by Aitoro et al. 2017 in a murine model revealed a direct correlation between the intake of butyrate and the suppression of acute allergic responses [62].

### 4.3. Lipoteichoic Acid (LTA)

**Figure 3:**
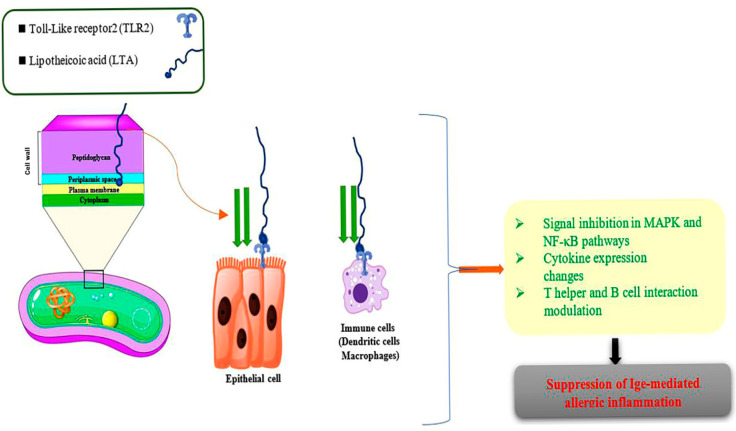
The main mechanisms of LTA against food allergies.

LTA constitutes a fundamental element of the cell wall in *Lactic acid bacteria* (*LAB*). It is classified as a specific type of teichoic acid that is covalently attached to a glycolipid within the cytoplasmic membrane. LTA is critically involved in mediating the host’s immune response. Moreover, the postbiotic effects of LTA modulate cytokine-mediated immune activities and may serve as a promising strategy for maintaining gut homeostasis in the face of excessive inflammation [63]. The postbiotics derived from *Lactobacillus plantarum* exhibit a downregulation of TNF-α-driven inflammatory cytokine transcription under in vitro conditions through a TLR-2-associated mechanism, leading to the inhibition of the NF-κB and Mitogen-Activated Protein Kinase signaling pathways [64]. Several significant mechanisms are implicated in the functional roles of LTA in the treatment of food allergies, which include: (1) the modulation of Treg cell activities Via HDAC inhibition, (2) the capacity to activate GPRs, particularly GPR43 and GPR109A, (3) serving as a primary energy source for intestinal epithelial cells, 4) promoting the synthesis of IL-10 and IFN-γ cytokines while reducing the rate of DNA methylation (Figure 3) [65].

## 5. The Dose–Response Effects of Postbiotics in FA

The dose–response relationships pertaining to postbiotics in the management of food allergies elucidate their capacity to modulate both immune and epithelial functionalities in a manner that is dependent on concentration, resulting in distinct outcomes across diverse types of food allergies. At optimal concentrations, postbiotics, which include short-chain fatty acids, microbial peptides, and cell wall components, enhance the integrity of the intestinal barrier, stimulate the functionality of regulatory T-cells, and inhibit pro-inflammatory cytokines, thereby reducing hypersensitivity reactions [66]. However, excessively high dosages may induce dysregulated immune activation or a loss of tolerance, thereby underscoring the critical importance of precise dosage calibration. Moreover, IgE-mediated food allergies, which are characterized by immediate hypersensitivity and mast cell degranulation, may derive enhanced benefits from postbiotics that stabilize epithelial barriers and attenuate IgE-mediated signaling pathways. In contrast, non-IgE-mediated food allergies, characterized by delayed T-cell-driven inflammatory responses, may demonstrate a superior response to postbiotics that elicit anti-inflammatory and tolerogenic effects through the modulation of dendritic cell activity and cytokine profiles. This variability highlights the necessity for individualized dosing strategies and personalized therapeutic interventions tailored to the specific type of food allergy and the underlying immune mechanisms involved [67].

## 6. Functional Foods-Based on Postbiotics in FA Treatment

Current therapeutic approaches for IgE-mediated food allergies predominantly emphasize the avoidance of identified allergens, alongside the administration of antihistamines and corticosteroid treatments, which are characterized by their limited efficacy and a range of adverse effects. Immunotherapy targeting food allergens is designed to induce desensitization and foster enduring immune tolerance through a systematic increase in exposure to these allergens [68]. Nonetheless, this intervention is associated with a considerable frequency of negative reactions and necessitates prolonged treatment duration. Consequently, there exists a pressing requirement to innovate alternative therapeutic strategies, such as functional foods. Metabolites synthesized by gut microbiota exert a profound influence on immune cells and gastrointestinal health. Recent empirical evidence indicates that certain postbiotics may facilitate intestinal homeostasis and mitigate food allergies [61]. The outcomes of recent investigations are particularly noteworthy, as they reveal that postbiotics exert a direct effect on mast cells by epigenetically modulating the signaling molecules linked to FcεRI [69]. Significantly, heightened concentrations of the postbiotics butyrate and propionate observed in fecal samples during early childhood are correlated with a reduced susceptibility to food allergies [70]. Novel discoveries underscore the crucial function of postbiotics in reinforcing epithelial barrier integrity, promoting oral tolerance, and offering protection against food allergies. This assertion is further corroborated by findings indicating that children diagnosed with cow’s milk allergy demonstrated diminished levels of fecal butyrate in comparison to healthy counterparts [71].

### 6.1. Kefir

**Figure 4:**
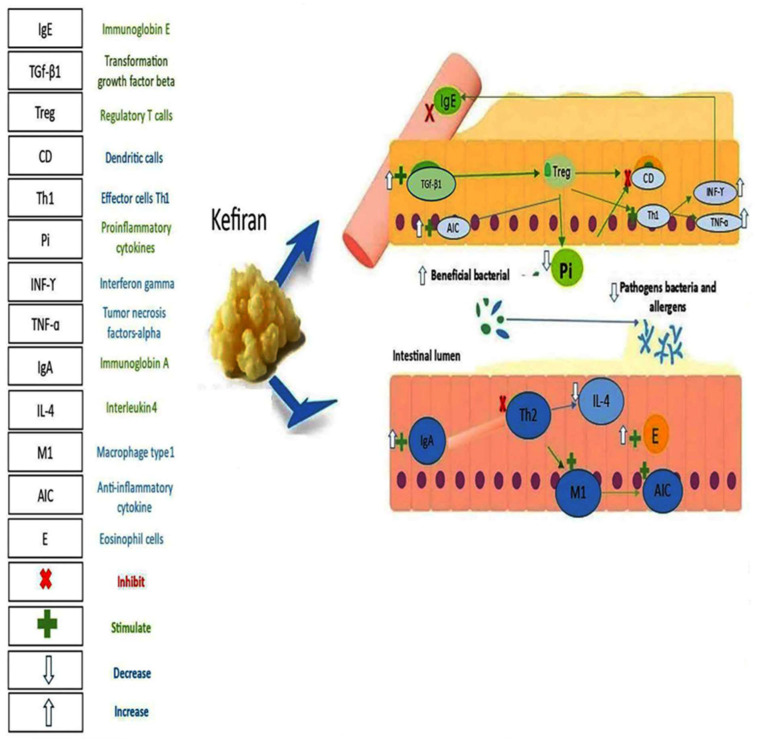
The benefits of kefiran on food allergy.

Kefir is recognized as a probiotic beverage resulting from the symbiotic fermentation of milk by LAB and yeasts encompassed within an exopolysaccharide-protein complex known as kefir grains. Kefiran represents a crucial element that contributes to both the viscosity and the health-promoting attributes of kefir. Its concentration within kefir is influenced by multiple variables, including the type of milk utilized, the duration of fermentation, temperature settings, and the specific microbial composition present. Generally, kefiran constitutes approximately 0.1–0.8% of the liquid portion of kefir, whereas it can represent 2–10% of the dry mass of kefir grains. Extended fermentation periods and favorable conditions are typically associated with enhanced production of kefiran, thereby augmenting the distinctive texture and functional characteristics of the beverage [72]. Unlike other fermented products, kefir is distinct in that it is derived from kefir grains, which encompass a specific and intricate assemblage of lactic acid and acetic acid-producing bacteria, as well as lactose fermenting and non-fermenting yeasts that coexist in a symbiotic association [34,73]. Throughout their growth phase, kefir microorganisms synthesize biopolymers that may be extruded beyond the bacterial cell wall, culminating in the formation of a heterogeneous array of substances referred to as EPS. This collection comprises polymers originating from the kefir microflora, specifically kefirans and dextran. These postbiotics possess the capability to interact with eukaryotic cells, modulate immune responses, and activate pertinent signaling cascades to promote gastrointestinal homeostasis, thereby augmenting the endogenous probiotics of each host rather than introducing unfamiliar strains [74]. Hence, the employment of postbiotics has garnered attention owing to its prospective role in enhancing immune tolerance and addressing food allergies without precipitating adverse effects. Furthermore, the application of postbiotics in the management of cow’s milk allergy appears promising, as it facilitates the development of kefir-like products utilizing non-dairy raw materials, given that kefiran can be extracted from the isolation of kefir grains [74]. Kefiran, the principal exopolysaccharide derived from kefir grains, along with yeasts and lactic-acid bacteria, constitutes the fundamental composition of kefir. From a chemical perspective, this polysaccharide comprises a repetitive branched unit of hexa- or hepta-saccharides, characterized by a consistent unit of pentasaccharides wherein one or two residues are randomly linked [75]. This polysaccharide consists of equal proportions of galactose and glucose, playing a pivotal role in the adhesion of microorganisms within kefir grains, thereby facilitating the attachment of the microbiota to the proliferating matrix. Notably, due to its chemical structure, kefiran is resistant to hydrolysis by the digestive enzymes present in the human gastrointestinal tract, rendering it non-absorbable; however, it can be fermented by bacteria within the large intestine [76]. These compounds play an important role in the immune system and are known to be a key factor in modulating allergic responses to food due to their potential to enhance epithelial barrier function and stimulate appropriate immune responses (Figure 4) [77,78].

### 6.2. Yogurt

With the burgeoning popularity of yogurt, manufacturers are incessantly investigating compounds possessing distinctive properties to formulate functional foods and entice consumers, which is a paramount objective for research and presents a significant challenge for both scientific inquiry and industrial practice. Given that yogurt serves as an exemplary source of vital nutrients, it has emerged as a prevalent option for fortification and is widely regarded as a healthful choice [79]. In recent years, entities within the food industry, encompassing both manufacturers and researchers, have persistently directed their efforts towards the utilization of high-nutritional-value compounds in the production of advantageous yogurt. Consequently, a substantial body of research has been disseminated concerning the augmentation of yogurt with a variety of compounds, inclusive of vitamins, minerals, fatty acids, prebiotics, probiotics, flavorings, and botanical extracts. The influences of these supplementary compounds on the physicochemical, sensory, textural, and microbial characteristics of fortified yogurt have been systematically assessed in comparison to traditional yogurt [80]. Nonetheless, the volume of investigations scrutinizing the implications of super healthy yogurts on human health remains markedly limited. Thus, owing to the significance of this topic, this review study will address the health ramifications associated with fortified yogurts, particularly those enriched with vitamins, bioactive compounds, and postbiotic substances [81].

Functional foods are defined as those that encompass beneficial compounds in addition to standard nutrients. One of the methodologies for generating health-promoting foods involves the incorporation of postbiotics into the food matrix [82]. Yogurt is recognized as one of the most appropriate mediums for delivering postbiotics, attributed to its diverse nutritional and therapeutic attributes, as well as its widespread popularity and acceptance among global consumers. Yogurts enriched with probiotics help improve health. However, the yogurt matrix may not be conducive to every type of postbiotic; therefore, additional research is warranted to identify the most suitable compounds [83]. Moreover, the concentration of postbiotics present in yogurt is influenced by several variables, including the specific bacterial strains employed, the duration of fermentation, and the conditions under which the product is stored. Generally, extended fermentation periods alongside the utilization of resilient probiotic strains lead to elevated concentrations of postbiotics, thereby enhancing the health-promoting attributes of yogurt. The precise concentration of these compounds can exhibit significant variability, indicative of disparities in production techniques and microbial dynamics. Nevertheless, it is important to note that the yogurt matrix may not be favorable for all varieties of postbiotics; consequently, further investigation is essential to pinpoint the most appropriate compounds [83].

Yogurt augmented with postbiotics contributes positively to the management of food allergies by supplying bioactive compounds that assist in modulating immune responses and promoting gastrointestinal health. Throughout the fermentation process, yogurt generates a variety of postbiotics, including short-chain fatty acids, peptides, and inactivated bacterial components, which retain their efficacy even after the viability of the live bacteria diminishes [84]. These substances are capable of fostering immune tolerance through the stimulation of regulatory T cells and the attenuation of pro-inflammatory cytokine activity associated with allergic responses. Furthermore, postbiotics found in yogurt aid in reinforcing the intestinal barrier, thereby restricting the translocation of allergens into the bloodstream and consequently mitigating the risk of immune sensitization. As a safe and stable functional food, yogurt enriched with postbiotics presents a supportive dietary strategy for the management of food allergies, particularly in vulnerable populations such as children and individuals with compromised immune systems [85].

### 6.3. Fermented Milk

The incorporation of fermented milk enriched with postbiotics has emerged as an intriguing functional aliment in the therapeutic management of food allergies, attributable to its capacity to modulate immune responses and enhance gastrointestinal health. Throughout the fermentation process, advantageous microorganisms such as *Lactobacillus* and *Bifidobacterium* spp. generate a diverse array of bioactive constituents, including SCFAs, peptides, exopolysaccharides, and inactivated microbial cellular components. In individuals afflicted with food allergies, these postbiotics may assume a pivotal function in attenuating allergic inflammation and fostering the establishment of immune tolerance to dietary antigens, a critical factor in diminishing hypersensitivity reactions. Empirical evidence from both preclinical and clinical investigations indicates that fermented milk containing postbiotics may possess the potential to diminish the allergenic properties of milk proteins and alleviate allergic responses. A specific In Vivo investigation utilizing a murine model of cow’s milk allergy revealed that fermented milk produced with *Lactobacillus helveticus* and *L. plantarum* considerably reduced levels of IgE, IgA, and IgG, alongside plasma histamine and mast cell protein-1. Additionally, it enhanced the immune equilibrium between Th1/Th2 and Treg/Th17 populations, augmented gut microbial diversity, and increased SCFA concentrations—indicating that postbiotic metabolites synthesized during fermentation can proficiently modulate immune and microbiota responses [86]. In humans, a randomized multicenter trial evaluated an infant formula containing heat-killed *Bifidobacterium breve C50* and *Streptococcus thermophilus*, which constitutes a postbiotic preparation in fermented milk. Compared to standard formula, infants receiving HKBBST (500 mL) had significantly fewer positive skin-prick tests to cow’s milk allergens (1.7% Vs. 12.5%, *p* = 0.03) and lower incidence of gastrointestinal and respiratory allergy-like events at 4, 12 and 24 months after birth. This suggests postbiotic-containing fermented milk can reduce early allergic sensitization and symptoms in high-risk infants [87]. Larger epidemiological findings further support a protective association between habitual intake of fermented milk products and allergy development. A study of students in Japan found that regular consumption of yogurt or fermented milk was associated with significantly lower total serum IgE levels and reduced prevalence of various allergic diseases compared to peers with low intake. Interestingly, no such association emerged for fermented soybean foods, indicating a potential specificity of dairy-based fermentation for allergy modulation [88].

### 6.4. Non-Dairy Sources

Dairy products possess inherent limitations that constrain the incorporation of postbiotics within their matrix. A primary drawback of dairy products in the context of food allergy management is their considerable propensity to elicit allergic responses in predisposed individuals. The proteins found in cow’s milk, notably casein and whey, rank among the most prevalent allergens, particularly among pediatric populations, and can elicit a spectrum of manifestations ranging from mild dermatological reactions to life-threatening anaphylaxis. Furthermore, even dairy derivatives that have undergone processing, such as cheese, yogurt, or butter, may retain allergenic proteins that present significant risks. Additionally, the ubiquitous inclusion of dairy as an ingredient in a myriad of packaged food items renders total avoidance challenging, thereby heightening the probability of inadvertent exposure [89].

Traditional non-dairy fermented foods constitute a vital dietary reservoir of postbiotics, which are bioactive compounds derived from microbial activity that persist post-fermentation and exhibit potential health advantages for individuals with food allergies. Noteworthy examples of traditional non-dairy fermented foods that yield these bioactive metabolites encompass vegetable-based ferments, such as kimchi, sauerkraut, and various pickled vegetables; legume-derived products, including tempeh, natto, and miso; and cereal-based preparations like sourdough bread, kvass, and fermented grain beverages prevalent in African or Asian cultures. The fermentation process not only augments flavor and extends shelf life but also produces postbiotic compounds that may assist in diminishing allergic sensitization through the promotion of immune tolerance and the stabilization of gut microbiota composition [90].

Within the sphere of food allergies, the significance of postbiotics sourced from traditional non-dairy fermented foods becomes particularly pronounced, as they can effectively serve as an alternative for individuals who are precluded from consuming dairy-derived probiotic products such as yogurt or kefir. Empirical evidence indicates that specific postbiotics, notably SCFAs such as butyrate and propionate, possess the capacity to modulate T-cell differentiation, enhance the activity of regulatory T-cells, and inhibit pro-inflammatory cytokines implicated in allergic responses. Furthermore, bioactive peptides and polysaccharides generated during the fermentation process may influence gut microbiota composition and bolster mucosal immunity, consequently mitigating intestinal permeability and averting allergen translocation [91]. By integrating non-dairy fermented foods into their dietary regimen, individuals with food allergies may experience enhancements in gut health and reductions in allergic reactions without the concomitant risk of exposure to dairy allergens. Therefore, traditional plant- and grain-based fermented foods emerge as a secure and functional source of postbiotics, presenting a natural dietary strategy to aid in the prevention and management of food allergies. The prospective capability of enhancing a diverse spectrum of non-dairy staple and avant-garde food products with purified or semi-purified postbiotic extracts is exceedingly optimistic, as it reconciles the intersection of nutrition, functionality, and consumer aspirations for sustainable health interventions [92]. In contrast to probiotics, postbiotics—comprising bioactive metabolites, peptides, cell wall fragments, and various microbial byproducts—exhibit inherent stability, thermal resilience, and a greater ease of integration into a multitude of food matrices without jeopardizing viability. This innovation creates avenues for their incorporation into non-dairy staples such as breads, cereals, plant-based beverages, and snacks, alongside novel functional foods aimed at promoting gut health, immunity, and metabolic regulation. Moreover, their integration can facilitate personalized nutrition paradigms, imparting health advantages to demographics with lactose intolerance, dairy allergies, or adherence to vegan lifestyles [93]. As ongoing research elucidates specific health-enhancing mechanisms, postbiotic-enriched products may materialize as a novel classification of functional foods, providing manufacturers with a reliable, versatile, and scientifically substantiated approach to augmenting everyday dietary practices. One of the principal technical obstacles in the application of postbiotics to non-dairy functional foods resides in their intrinsic sensory and formulation complexities. Numerous postbiotic extracts encapsulate peptides, short-chain fatty acids, or cell wall constituents that may impart bitter, sour, or umami-like off-flavors, which can adversely influence consumer acceptance in staple or indulgent products. This situation necessitates the implementation of effective flavor masking techniques, such as microencapsulation, complexation with natural carriers, or the utilization of flavor modulators, to maintain palatability [94]. A further significant challenge involves ensuring dosage consistency, particularly in solid or semi-solid matrices like baked goods, bars, or cereals, where uneven distribution could undermine both efficacy and regulatory adherence. The stability under processing conditions—such as elevated temperatures during extrusion or baking—also constitutes a challenge, necessitating the development of tailored delivery systems that safeguard postbiotic integrity while preserving functional activity [95].

Simultaneously, these challenges present avenues for innovation in formulation science and product development. Progress in encapsulation technologies, including lipid-based systems, spray-drying, or nanoemulsions, can bolster stability, mask undesirable flavors, and enable precise dosage regulation. Non-dairy food categories—comprising plant-based beverages, protein powders, savory snacks, and fermented grain products—furnish adaptable platforms for the targeted delivery of postbiotics to health-oriented consumers. Furthermore, the compatibility of postbiotics with prevailing clean-label and plant-based trends creates opportunities to distinguish products within a competitive landscape, particularly when associated with clinically validated health claims [96]. By harnessing both biotechnological advancements in postbiotic purification and food engineering methodologies for incorporation, manufacturers can surmount technical impediments and unlock the comprehensive potential of postbiotics in defining the forthcoming generation of functional foods. Another aspect that should be considered is the synergistic role of postbiotics with other bioactive compounds such as prebiotics (Fructooligosaccharides) in the treatment of food allergies [97].

## 7. The Synergistic Effects of Postbiotics with Prebiotics

The integration of postbiotics and prebiotics constitutes a synergistic approach for the management of food allergies by addressing both immune modulation and the support of gut microbiota. Prebiotics, including inulin, galacto-oligosaccharides, and fructo-oligosaccharides, selectively promote the proliferation and function of beneficial intestinal bacteria, resulting in the augmented synthesis of metabolites such as SCFAs. Postbiotics, which comprise these metabolites along with various microbial constituents, can be administered directly to enhance anti-inflammatory responses and bolster barrier-protective mechanisms. When utilized in conjunction, prebiotics stimulate the host microbiota to autonomously produce postbiotic substances, whereas the supplementation of postbiotics guarantees the immediate and consistent delivery of bioactive molecules [35]. This dual strategy fortifies intestinal barrier integrity, mitigates allergen translocation, and fosters the activation of regulatory T-cells, thereby facilitating the restoration of immune tolerance to dietary antigens. In a synergistic manner, the combination of postbiotics and prebiotics has the potential to attenuate allergic responses more efficaciously than either component utilized in isolation, by simultaneously targeting both the underlying causes and resultant effects within the gut-immune axis. Prebiotics foster a conducive microbial environment that consistently generates advantageous postbiotic metabolites, whereas postbiotics engage in direct anti-allergic mechanisms, such as the downregulation of IgE-mediated responses and the modulation of cytokine profiles. This synergistic effect may hold particular significance in interventions during early life, when the development of gut microbiota is paramount for immune system programming and the mitigation of allergic diseases [98]. Through the stabilization of gut homeostasis, the reduction in inflammation, and the facilitation of long-term oral tolerance, the interplay between postbiotics and prebiotics presents a promising nutritional and therapeutic strategy for the management of food allergies Via functional foods and customized dietary interventions. Beyond the synergistic effects of postbiotics in conjunction with other compounds, forthcoming research endeavors may elucidate additional facets of the role of postbiotics in the therapeutic management of food allergies [99].

## 8. Future Prospects for Functional Food-Based on Postbiotics

In the foreseeable future, advancements in molecular biology and metabolomics may facilitate the identification of distinct postbiotic constituents that exert direct effects on allergy-related pathways, including the modulation of IgE-mediated immune responses. Food manufacturers may integrate these specific compounds into commonplace products, ranging from dairy alternatives to snack bars, thereby providing individuals with allergies with accessible means to enhance their immune resilience [50]. Personalized nutrition, underpinned by genetic and microbiome profiling, could empower consumers to select postbiotic-enriched foods customized to their individual allergy profiles, further augmenting the effectiveness and consumer confidence in these products. Regulatory frameworks are anticipated to evolve as well, establishing more precise guidelines for the labeling and substantiation of health claims, which will contribute to the establishment of credibility within this burgeoning market [100,101]. From a commercial perspective, postbiotic-based functional foods may experience swift adoption as public awareness of the connections between gut and immune health increases, coinciding with the rising incidence of food allergies on a global scale. These products may also attract consumers who are inclined toward preventive solutions instead of reactive treatments, particularly parents of children who are predisposed to developing allergies [71,102]. The collaboration among food scientists, immunologists, and clinical researchers will be imperative to generate substantial evidence that supports specific postbiotic formulations. As the research foundation expands, one can anticipate the emergence of innovative product lines from fortified beverages to infant formulas positioned not merely as nutritional options but also as functional interventions in the management of allergies, which has the potential to revolutionize societal approaches to allergic diseases [98,103].

## 9. Conclusions

Functional-based postbiotics signify a potentially effective adjunctive approach in the prophylaxis and management of FAs. By administering bioactive microbial metabolites devoid of the risks linked to live microorganisms, they provide a secure, stable, and targeted methodology for the modulation of immune responses. Empirical evidence indicates that postbiotics may enhance gut barrier integrity, regulate inflammatory pathways, and facilitate the establishment of oral tolerance through mechanisms such as the increased synthesis of SCFAs, modulation of dendritic cell functionality, and restoration of a healthy microbiota-derived metabolic profile. Although preclinical and preliminary clinical investigations yield promising results, further extensive, rigorously controlled human trials are imperative to optimize formulations, ascertain effective dosages, and elucidate long-term safety. Ultimately, the incorporation of functional-based postbiotics into dietary interventions could potentially pave the way for innovative, personalized, and more tolerable therapeutic alternatives for individuals afflicted with FAs.

## Figures and Tables

**Table 1:** The difference between probiotics and postbiotics.

Aspect	Probiotics	Postbiotics	References
Definition	Live microorganisms that confer health benefits when consumed in adequate amounts.	Non-living microbial cells, components, or metabolites that provide health benefits.	[1]
Nature	Living, self-replicating organisms.	Non-living, inanimate compounds.	[2]
Examples	*Lactobacillus*, *Bifidobacterium*, *Saccharomyces boulardii*.	SCFAs, cell wall fragments, peptides, enzymes.	[3]
Mechanism of Action	Colonize the gut, compete with pathogens, produce beneficial metabolites, modulate immunity.	Act directly through bioactive compounds, modulating immunity, inflammation, and gut barrier.	[4]
Stability	Sensitive to heat, pH, oxygen, and storage conditions.	More stable; longer shelf life; unaffected by conditions that kill live microbes.	[5]
Safety	Risk of infection in immunocompromised individuals.	Safer; no risk of infection since non-living	[6]
Source	Fermented foods (yogurt, kefir, kimchi) and supplements.	Derived from probiotic fermentation byproducts or heat-killed probiotics.	[7]
Delivery Requirement	Must survive stomach acid and bile to reach the gut alive.	Do not need to survive digestion; already bioactive.	[8]
Clinical Evidence	Well-studied for digestive health, immunity, IBS, antibiotic-associated diarrhea.	Emerging evidence for immunity, anti-inflammatory effects, gut barrier support.	[9]
Individual Variability	Effects vary with strain, dose, and host microbiome.	Less dependent on host microbiome; effects are more consistent.	[10]
Regulatory Status	Regulated as supplements or functional foods; strain-specific claims.	Less regulated; definitions still evolving in many countries.	[11]
Use Cases	Restoring gut microbiota, supporting digestion, preventing infections.	Complementing probiotics, reducing inflammation, immune modulation.	[12]
Limitations	Require careful storage, strain-specific effects, possible side effects.	Less diverse effects than probiotics; research still growing.	[13]
Synergy	Can be combined with prebiotics (synbiotics) and postbiotics.	Can complement probiotics or replicate some of their effects.	[13]

## Data Availability

The data that support the findings of this study are available from the corresponding author upon reasonable request.
